# A prospective cohort conversion study of twice-daily to once-daily extended-release tacrolimus: role of ethnicity

**DOI:** 10.1186/2047-1440-3-7

**Published:** 2014-03-10

**Authors:** Lauren Glick, Fernanda Shamy, Michelle Nash, Ahmed Sokwala, Tushar Malavade, GV Ramesh Prasad, Jeffrey S Zaltzman

**Affiliations:** 1 Keenan Summer Student Research Program, St. Michael’s, 30 Bond St, Toronto, Ontario M5B21W8, Canada; 2 Transplant Program, St. Michael’s, 30 Bond St, Toronto, Ontario M5B1W8, Canada; 3 University of Toronto Nephrology Training Program at St. Michael’s, 30 Bond St, Toronto, Ontario M5B1W8, Canada; 4 Department of Medicine, Division of Nephrology, Keenan Research Institute, St. Michael’s, 30 Bond St, Toronto, Ontario M5B1W8, Canada

## Abstract

**Background:**

Tacrolimus is a widely used calcineurin inhibitor in kidney transplantation. It is available as twice-daily Prograf® (Tac-BID) and once-daily Advagraf® (Tac-OD). Although therapeutically equivalent, some patients require dose adjustments to achieve similar trough concentrations [*C*_0_] after conversion. Tacrolimus exposure is affected by ethnicity in the *de novo* setting but the role of ethnicity in determining dose requirements and adjustments after conversion is unknown.

**Methods:**

In this study, 496 renal transplant recipients (RTRs) were prospectively converted from Tac-BID to Tac-OD, with dose adjustments targeted to achieve similar [*C*_0_] at 12 months post-conversion. Renal function, acute rejection and Tac dose adjustments by ethnicity were analyzed.

**Results:**

There were similar numbers of recipients from living and deceased donors. The mean transplant duration was 7 years. Of the RTRs, 60% were Caucasian and 40% were identified as belonging to an ethnic minority. There was no change in estimated renal function (eGFR) post-conversion to Tac-OD. At 12 months, 35/488 (7%) RTRs were receiving a reduced dose, 101/488 (21%) required a dose increase of which 77 (16%) were receiving at least a 30% increase in dose over baseline. The percentage of those in ethnic groups requiring a dose increase of >30% varied from 8.0% for South Asians to 27.5% for East Asians (*P* = 0.03), despite East Asians having a similar baseline dose of Tac-BID (3.59 mg/day) compared to the entire cohort (3.53 mg/day).

**Conclusions:**

Ethnicity may play an important role in dosing requirements when converting from Tac-BID to Tac-OD, unrelated to baseline dose. Further investigation is required to determine the reasons for ethnic variability when patients are converted between tacrolimus preparations.

## Background

Tacrolimus is a widely used calcineurin-inhibitor in kidney transplantation. It is available as both twice-daily Prograf® (Tac-BID) and once-daily extended-release Advagraf® (Tac-OD) (Astellas Pharma Inc, Tokyo, Japan). While considered therapeutically equivalent [1,2], in some patients conversion requires dose adjustments to achieve similar trough concentrations [*C*_0_] [3]. Our previously reported *de novo* experience indicated that non-Caucasians required significantly higher doses of both Tac-BID and Tac-OD to achieve equivalent serum concentrations [4]. However, in that study the Tac-BID and Tac-OD populations were separated by era owing to our program’s protocol switch from the former to the latter. The effect of ethnicity on intra-patient dosing requirements has not been previously reported. The purpose of this prospective cohort study, therefore, was to assess if ethnicity is an important determinant of dosing requirements in a population of stable renal transplant recipients (RTRs) undergoing conversion from Tac-BID to Tac-OD.

## Methods

The Greater Toronto Area has a population in excess of 5 million of which 43% identifies themselves as a visible minority and 50% are foreign-born [5]. All stable RTRs receiving Tac-BID were converted to Tac-OD in January 2012 due to concern about the potential for patient confusion resulting from the uncontrolled switching of tacrolimus preparations by retail pharmacists. About 500 stable RTRs, representing the entire Tac-BID population, were invited by appointment to initiate the switch. A transplant pharmacist and nurse provided the rationale for conversion and other necessary education including the need for [*C*_0_] monitoring at 1 and 4 weeks post-conversion. The St Michael’s Research Ethics Board approved the study.

### Conversion

Patients were converted from Tac-BID to Tac-OD on a 1 mg:1 mg basis for the total daily dose unless their most recent [*C*_0_] on Tac-BID was ≤3.0 ng/ml, in which case their Tac-OD dose was increased by 1 mg/day. Blood samples to measure [*C*_0_] and renal function were obtained at 1 week, 4 weeks and every 3 months post-conversion. As per the protocol, the goal was to maintain similar [*C*_0_] levels post-conversion. Changes in medication that could affect tacrolimus levels were monitored.

### Determination of ethnicity

Patients were classified by ethnicity based on the patient’s self-report and/or pre-transplant assessment records [6] as follows:

• Caucasian: any ancestry from Europe

• African Canadian: any ancestry from Africa either directly or via the Caribbean

• East Asian: ancestry from China, Mongolia, Japan, North or South Korea, Taiwan, Myanmar, Thailand, Laos, Cambodia, Vietnam, Malaysia, Singapore, Indonesia, or the Philippines

• South Asian: ancestry from India, Pakistan, Bangladesh, Sri Lanka, Nepal, Maldives, or Bhutan

• Middle Eastern: ancestry from Egypt, Algeria, Syria, Iraq, Iran, Lebanon, Jordan, Oman, Kuwait, Sudan, Tunisia, Morocco, Saudi Arabia, Yemen, UAE, Libya or the Palestinian Territories

• Other: used for various ethnicities present in low numbers and not otherwise included above

If a patient had mixed ancestry, the patient’s self-report was used for assignment to a single category.

### Outcomes

The primary outcome was to determine the frequency of RTRs who at last follow-up post-conversion, required >30% increase in Tac-OD compared to Tac-BID to achieve a similar [*C*_0_]. Secondary outcomes included the frequency of dose increase amongst various ethnic groups, renal function as assessed by the modification in diet renal disease equation (eGFR) and acute rejection rates post-conversion.

### Data and statistical analysis

This was a prospective cohort study with patients entering at the time of conversion from January 2012 through May 2013, with analysis in July 2013 (3 to 18 months follow-up).

Patient demographics, type of transplant (living versus deceased), time since transplant and ethnicity were collected, in addition to Prograf dosage and [*C*_0_] at the time of conversion and Advagraf dosage and [*C*_0_] post-conversion as previously described. The data on renal function by serum creatinine(Cr) and eGFR were collected for 6 months pre-conversion to 18 months post-conversion. However, the primary analysis of outcomes of interest was at 12 months post-conversion. Adverse events including episodes of biopsy-proven acute rejection were also recorded. The mean and standard deviation (SD) of Tac dosages were collected, and the frequency of dose increases between ethnic groups was analyzed using a chi-square test. Renal function pre- and post-conversion was compared using ANOVA.

## Results

Between January 2012 and May 2013, 496 RTRs were converted from Tac-BID to Tac-OD. Of the cohort, 90% were on prednisone and 76% were also taking either enteric-coated mycophenolic acid or mycophenolate mofetil. Post-conversion, there were no changes in medication known to interact with tacrolimus, such as diltiazem. There were no changes in any concomitant immunosuppressive agents. At the time of the last follow-up there were no allograft losses, and no episodes of acute rejection were noted. In the evaluation of renal function, eight patients were excluded from final analysis based on serum creatinine at time of conversion >250 μmol/l. These patients were excluded so as not to confound renal function outcomes based on conversion in patients with expected deteriorating renal function prior to conversion. Therefore, 488 patients were included in the renal function and tacrolimus dose adjustment analysis. The baseline demographics are outlined in Table 1. As shown, there were similar numbers of recipients with living and deceased donors, the mean transplant duration was 7 years, 56.8% of the RTRs were Caucasian and 40% were identified as belonging to an ethnic minority cohort.

**Table 1:** **Baseline demographics: *N*** **= 496**

**Parameter**	**Value**
Age, mean (SD)	55 (12)
Male, *N* (%)	303 (61)
Deceased donor	249
Living donor	247
Transplant duration, years (range)	7 (0.8 to 14)
Ethnicity, *N* (%)	496 (100)
Caucasian, *n* (%)	282 (56.8)
East Asian, *n* (%)	91 (18.3)
South Asian, *n* (%)	75 (15.1)
African Canadian, *n* (%)	18 (3.6)
Middle Eastern, *n* (%)	12 (2.4)
Other, *n* (%)	10 (2.0)

**Figure 1:**
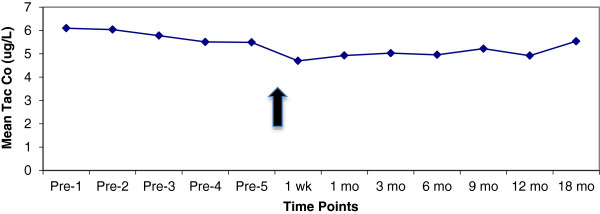
**Mean tacrolimus trough concentration [*C***_**0**_**] (ng/ml), pre- and post-conversion.** Arrow indicates time of conversion.

**Figure 2:**
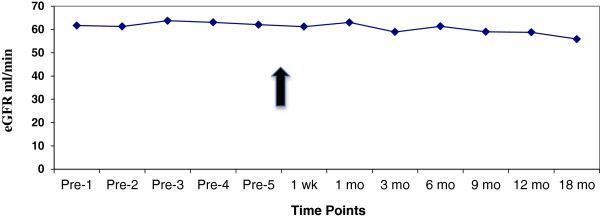
**Mean renal function by MDRD eGRF (ml/min), pre- and post-conversion.** Arrow indicates time of conversion.

As shown in Figure 1, at the time of conversion mean (SD) [Tac-BID] was 5.5 (2.3) ng/ml. At one week and one month post-conversion, [Tac-OD] decreased to 4.7 (2.0) and 4.9 (2.1) ng/ml respectively, necessitating an increased dose titration as per the protocol. By 9 months post-conversion, [Tac-OD] was 5.2 (2.4) ng/ml. To maintain a stable [tacrolimus] there were on average 0.27 dose adjustments per patient (range: 0 to 4). Of note, by 12 months post-conversion 35/488 (7%) RTRs were receiving a reduced dose, whereas 101/488 (21%) required a dose increase, of which 77 (16%) were receiving >30% increase in dose over the baseline (Table 2). As further illustrated in Table 2, the percentage of patients requiring a dose increase of 30% or greater varied from 8.0% for South Asians to 27.5% for East Asians, the latter being statistically significant (*P* = 0.03) by chi-square analysis. We could not find any relation between initial dose and the dose increase for either the entire cohort or ethnic-specific groups. As illustrated in Figure 2, there was no change in renal function as estimated by eGFR post-conversion.

**Table 2:** Conversion data at 12 months by ethnicity

**Conversion**	***N***	**Mean (SD) Tac-BID dose (mg/day)**	**Mean (SD) [Tac] bid (ng/ml)**	**Mean (SD) Tac-OD dose (mg/day)**	**Mean (SD) [Tac] OD (ng/ml)**	**Percentage of cohort requiring >30% increase in Tac-OD dose**
Total	488	3.53 (2.01)	5.49 (2.36)	3.75 (2.03)	5.15 (2.48)	15.7
Caucasian	282	3.23 (1.94)	5.58 (2.34)	3.42 (2.14)	5.48 (2.93)	13.5
East Asian	91	3.59 (2.27)	5.46 (2.35)	4.07 (2.28)	4.64 (1.38)	27.5*
South Asian	75	3.67 (2.25)	5.00 (1.62)	3.86 (2.31)	4.54 (1.38)	8.0
African Canadian	18	6.18 (3.87)	5.13 (1.43)	6.23 (4.20)	5.92 (2.33)	11.1
Middle Eastern	12	3.04 (1.76)	6.39 (3.26)	2.96 (1.57)	4.70 (2.30)	8.3
Other	10	2.89 (2.33)	5.42 (2.37)	2.06 (2.14)	5.40 (2.31)	20.0

*P = 0.03 compared to all other cohorts.

BID, twice daily; OD, once daily; SD, standard deviation; Tac, tacrolimus.

## Discussion

We report on the first observational cohort trial that demonstrates clear differences in tacrolimus dosages following conversion from Tac-BID to Tac-OD based on ethnicity. Almost 500 RTRs served as their own controls in this study, the second largest single-center conversion study reported to date.

The potential benefit of Tac-OD versus Tac-BID is the potential for increased patient adherence. A number of studies have described experiences with the conversion from Tac-BID to Tac-OD. The initial recommendation of conversion from Tac-BID to Tac-OD was 1 mg:1 mg, based upon pivotal studies in stable RTRs [1]. However, with increasing experience, most groups have reported that upon conversion, tacrolimus exposure, measured by either *C*_0_ or area under the curve, is reduced in some but not all RTRs [3,7-9]. Others have explored the relation with the *CYP3A5* genotype, finding dose increases upon conversion in those recipients with *CYP3A5* *1/*3 or *CYP3A5* *1/*1 compared to no increase in recipients with the *CYP3A5* *3/*3 [9-11]. Slatinska *et al*. described the largest single-center conversion study involving 589 RTRs. Although not reported, it is assumed by readers that the majority of these RTRs were Caucasian [12].

The renal transplant program at St Michael’s in Toronto, Canada, has one of the world’s most ethnically diverse populations, comprising almost 50% new immigrants and 43% self-identified as a visible minority. This allowed us to study prospectively dose adjustments following conversion from Tac-BID to Tac-OD, based on ethnicity. It has been well established that amongst some ethnic cohorts, such as African Americans, higher doses of tacrolimus regardless of formulation are required, because of their faster metabolism owing to a higher prevalence of *CYP3A5* *1/*3 and *1/*1 and perhaps other yet unidentified polymorphisms [13]. This was also observed in our Toronto-based population with African Canadians requiring an average 6.18 mg/day compared to 3.53 mg/day for the entire cohort (*P* = 0.004). However, only 11% of this group of ‘fast metabolizers’ as a whole, required an increase in Tac-OD following conversion.

Our unique observation is that upon conversion, dose increases were required with increased frequency in distinctive ethnic groups. Of almost 500 RTRs, 15.7% required a significant dose increase to maintain equal *C*_0_ [Tac]. However amongst RTRs of East-Asian origin, 27.5% required a significant increase in dose upon conversion from Tac-BID to Tac-OD. As stated earlier, there may be a relation between being a so-called *CYP3A5* expresser (*1/*1 or *1/*3) and the requirement for increased tacrolimus doses on conversion from Tac-BID to Tac-OD. However, these genotypes are not believed to be more prevalent in the East Asian population. Of interest, in a pediatric conversion study from Korea, 44% of the population required a dose increase while 26.5% required a decrease following conversion [7]. Niioka *et al*. reported 25% lower 24-hr area-under-the-curve values in a conversion study involving Japanese RTRs, although this was seen mainly in those with the *CYP3A5* *1/*3 genotype (15% of their cohort) [9]. Thus there may be other yet unidentified genetic polymorphisms that have a role for certain ethnic groups. Unlike the African Canadian cohort, the baseline tacrolimus dosages for the East Asian population were no different from the entire cohort (3.59 mg/day versus 3.53 mg/day).

With regard to renal function, we did not find any difference in eGFR on conversion from Tac-BID to Tac-OD. Although two groups have reported an improvement in renal function following conversion [14,15], this may have been due to reduced tacrolimus exposure following conversion. A multi-center conversion study of over 1,800 RTRs did not find any renal function change following conversion [16]. While there may be a renal function advantage with once daily dosing, owing to a lower single peak exposure compared with Tac-BID, more sensitive tests of GFR are likely needed to demonstrate this benefit if it exists [17].

## Conclusions

The present study confirms that conversion from Tac-BID to Tac-OD necessitates an increase in dosage for some RTRs. The magnitude of this increase is affected by ethnicity, particularly for East Asians. Since genetic polymorphism testing is not practical for most transplant programs and unlikely to be cost effective if employed on a population-wide basis, ethnicity should be considered in determining the initial post-conversion Tac-OD dose. Nonetheless, pharmacogenetic and pharmacokinetic investigations deserve consideration to better delineate optimal conversion strategies.

## Abbreviations

BID: twice daily; OD: once daily; RTR: renal transplant recipient; SD: standard deviation; Tac: tacrolimus.

## Competing interests

JSZ and GVRP have previously received funding from Astellas Canada. JSZ has also received funding from Astellas Global. LG received a Keenan Summer Student Bursary from St Michael’s Hospital for her work on this project. There are no other conflicts of interest to declare.

## Authors’ contributions

LG collected and analyzed the data. FS monitored tacrolimus levels post-conversion. AS and TM collected the data. NM analyzed the data. GVRP managed the patients and helped to write the manuscript. JSZ was the senior author. JSZ conceived the study, analyzed the data, supervised some of the other authors and wrote the manuscript. All authors read and approved the final manuscript.
